# Liquid nitrogen-based cryoablation: complication rates for lung, bone, and soft tissue tumors cryoablation

**DOI:** 10.1093/bjr/tqae171

**Published:** 2024-09-03

**Authors:** Franco Orsi, Aida Shazlin Hamiddin, Caterina Sattin, Caterina Pizzi, Gianluca Maria Varano, Paolo Della Vigna, Giovanni Mauri, Daniele Maiettini, Guido Bonomo

**Affiliations:** Department of Interventional Radiology, Istituto Europeo di Oncologia (IRCCS), Milan, Via Giuseppe Ripamonti 435, Milan-20141, Italy; Department of Interventional Radiology, Istituto Europeo di Oncologia (IRCCS), Milan, Via Giuseppe Ripamonti 435, Milan-20141, Italy; Selayang Hospital, Selangor, Malaysia; Università degli studi di Milano, Milan, 20122, Italy; Università degli studi di Milano, Milan, 20122, Italy; Department of Interventional Radiology, Istituto Europeo di Oncologia (IRCCS), Milan, Via Giuseppe Ripamonti 435, Milan-20141, Italy; Department of Interventional Radiology, Istituto Europeo di Oncologia (IRCCS), Milan, Via Giuseppe Ripamonti 435, Milan-20141, Italy; Department of Interventional Radiology, Istituto Europeo di Oncologia (IRCCS), Milan, Via Giuseppe Ripamonti 435, Milan-20141, Italy; Department of Interventional Radiology, Istituto Europeo di Oncologia (IRCCS), Milan, Via Giuseppe Ripamonti 435, Milan-20141, Italy; Department of Interventional Radiology, Istituto Europeo di Oncologia (IRCCS), Milan, Via Giuseppe Ripamonti 435, Milan-20141, Italy

**Keywords:** Cryoablation, Liquid nitrogen, Interventional oncology

## Abstract

**Objective:**

This study aimed to assess the complication rate during and 24 hours after cryoablation in lung, bone, and soft tissue tumors.

**Methods:**

We reviewed complications in a total of 85 consecutive patients who underwent cryoablation using a liquid nitrogen-based system in various lesions between April 2017 and October 2022. There were no liver and renal lesions. Complications were categorized using the Society of Interventional Radiology classification.

**Results:**

Eighty-five patients were treated for 96 lesions in the bone (36.4%; 35 of 96), lung (18.8%; 18 of 96), and soft tissue (44.8%; 43 of 96). The primary technical success rate was 97.7% (83 of 85). The total grade 2 and 1 complication rates were 5.2% (5/96) and 20.8% (20/96), respectively. Two patients had asymptomatic pulmonary embolisms incidentally noted at the 24-hour follow-up computed tomography (grade 2). The most frequent complications were simple and hemorrhagic pleural effusions (18.7%; 18 of 96). Lung procedures had the highest complication rate, where 13 patients (72.2%; 13 of 18) reported complications, including 2 cases of symptomatic hydropneumothorax requiring drainage (grade 2) and an additional 2 days of hospital stay. Eight patients (24.2%; 8 of 33) with bone lesions and 4 (9.3%; 4 of 43) with soft tissue lesions experienced complications.

**Conclusion:**

Cryoablation using a liquid nitrogen-based system is safe, with only minor complications observed.

**Advances in knowledge:**

This study provides data on the safety of liquid nitrogen-based percutaneous cryoablation in tumors located in lung, in bones and in soft tissues. Despite using larger diameter cryoprobes than those typically reported with argon-based system, our experience shows that complications are mostly low and comparable in frequency and severity.

## Introduction

Interventional radiology is now the fourth pillar of the oncology field, alongside clinical oncology, surgical oncology, and radiation therapy.^1^^,^^2^ While surgery remains the standard of care for early-stage tumors, there is a growing role for ablative techniques in managing oncology patients, particularly those with multiple comorbidities or those who wish to avoid traditional surgery.^3^

For lung and soft tissue lesions, percutaneous cryoablation, thermal (heat) ablation, and stereotactic body radiation therapy, have recently been used as an alternative to surgery to minimize invasiveness.^2^^,^^8^^,^^21^^,^^22^ The most common bone lesions treated in interventional oncology are metastatic bone lesions, which can lead to skeletal-related events, such as pathological fractures, hypercalcemia, metastatic spinal cord compression, and bone pain.^5^^,^^10^^,^^19^^,^^20^ These conditions are usually associated with decreased physical function and quality of life.^19^ Both percutaneous cryoablation and thermal ablation have been demonstrated to be safe and effective minimally invasive treatments for palliating painful bone metastases, reducing the risk of skeletal-related events.^5^^,^^10^ It has become a well-established treatment modality and is widely used as a treatment for bone tumors in patients who cannot undergo surgery or in whom radiation therapy has failed^10^

Percutaneous cryoablation has been demonstrated to be a safe, minimally invasive treatment in oncology.^4–6^ It is based on using very low temperatures to ablate various lesions, including those in the lung, bone, breast, soft tissue, liver, kidney, and prostate.^7–14^ The iceball formed can be monitored with imaging during the procedure, allowing greater control of the ablation zone, a distinct advantage over other ablation modes such as radiofrequency, microwave, High Intensity Focused Ultrasound (HIFU), and irreversible electroporation.^15^^,^^16^ Various cryoablation systems employing different cryogenic materials, such as liquid nitrogen and argon gas, have demonstrated effectiveness in treating breast cancer.^11^

However, distinctions between these systems in terms of their settings and specifications are important.

Single cryoprobe systems utilizing liquid nitrogen may be performed under local anesthesia, making them particularly suitable for office-based treatments. Conversely, multiple cryoprobe systems employing argon typically necessitate general anesthesia or intravenous sedation and are less suitable for office-based procedures.^4^^,^^17^ During freezing, the tissue's water transitions from liquid to solid, forming an iceball. While multiple cryoablation systems may permit faster treatment times and treatment of larger lesions (inserting multiple adjacent cryoprobes), liquid nitrogen devices generally create larger ice balls and more rapidly than argon gas based devices,^5^^,^^9^ especially in a warm environment (body temperature).^4^^,^^18^ The liquid nitrogen-based cryoablation system has the advantage of small insulated flask storage ready for use, while high-pressure argon and helium gases are stored in large gas cylinders, requiring dedicated storage space and trained personnel for transport.^4^^,^^8^^,^^18^ Additionally, as rare noble gases, argon and helium (used for the thawing stage) are relatively costly in addition to the higher treatment costs (ie, multiple cryoprobes).

Cryoablation systems based on argon gas also requires a specially equipped room with approved safety mechanisms.^4^^,^^8^ It requires 300 bar large gas cylinders and validation by the institution's security department, restricting its general use.^4^ Cryoablation using a liquid nitrogen-based system was developed in 2006 (IceSense 3, IceCure Medical Ltd, Caesarea, Israel) and is approved by the USA Food and Drug Administration and CE certified for various indications. The liquid nitrogen-based system is cheaper than the argon gas-based one and easier to manage.^4^^,^^8^ It has been reported that cryoablation using a liquid nitrogen-based system provides a large ablation zone, usually using a single cryoprobe, potentially reducing the risk of complications compared to the multiple cryoprobes needed for the argon system.^4^^,^^8^ However, for some lesions, a large diameter cryoprobe (10 gauge) is used for the liquid nitrogen-based system, which might hypothetically cause more complications.

While the use of the liquid nitrogen-based system has been reported for the treatment of fibroadenoma and lung malignancies,^4^ its safety for the treatment of various other organs has not yet been well reported.

This study aimed to assess the complication rate during and 24 hours after cryoablation of different tumors in various organs. The primary technical success rate was also evaluated.

## Material and methods

This study was approved by the local ethical institutional review board and an informed consent was obtained from all individual participants.

From April 2017 to October 2022, this retrospective descriptive study reviewed complications observed in 85 patients who underwent cryoablation using a liquid nitrogen-based system in our institution. Patients with various lesions (benign or malignant) were included for treatment with either curative or palliative intent. Liver and renal tumors were not included in this study due to lack or limited data. No cryoblation procedures were performed in patients with liver tumors, and the number of patients with kidney tumors who underwent cryoblation was insufficient for inclusion in the study.

### Cryoablation procedure and technique

The decision for percutaneous cryoablation was made in a multidisciplinary meeting involving surgeons, oncologists, radiation therapists, pathologists, diagnostic, and interventional radiologists, with the intent of either curative (tumor reduction/elimination) or palliative (analgesic/palliative) treatments. All procedures were performed on an in-patient basis under general anesthesia by experienced anesthesiologists. Six interventional radiologists with more than 5 years of experience in percutaneous ablations performed the procedures. The IceSense3 cryoablation system (IceCure Medical Ltd Caesarea, Israel), with 3.4 mm (10 gauge) or 2.4 mm (13 gauge) cryoprobes, was used in all procedures. The size, number, and type of cryoprobes were selected according to the pre-operative imaging (CT, MRI, and US) assessment of tumor size, shape, and location, and also according to the therapeutic intent (curative or palliative). When a larger ablation zone was planned, up to three co-axial cannulas were inserted, spaced at least 2 cm apart, to cover the whole target. A single cryoprobe was then sequentially inserted through them to achieve a synergistic effect.

When needed, a percutaneous biopsy of the target lesion was performed before or during the procedure (through the co-axial cannula). For curative intent ablation, we established a projected isotherm of <−20°C, considering −20°C as a cutoff for the killing zone^8^^,^^14^^,^^17^^,^^23–25^ with a peripheral margin between 5 mm and 10 mm. Additional data are given in the Supplementary material.

### Follow-up

Immediate post-cryoablation and 24 hours after the procedure, a contrast-enhanced CT was always performed. Patients were then followed up clinically and with repeated CT at 4-6 weeks and every 3 months for the first year and annually thereafter.

### Complication

Complications during the cryoablation procedure and in the following 24 hours were assessed and recorded. Complications were categorized using the Society of Interventional Radiology classification.^26^^,^^27^ The adverse events (AEs) were defined as grade from 1 to 5 according to SIR classification (ie, grade 1 for mild AE, grade 2 for moderate AE, grade 3 for severe AE, grade 4 for life-threatening or disabling event and grade 5 in case of patient death or unexpected pregnancy abortion).^26^

### Technical success

Primary technical success was assessed and defined according to standard terminology as reported by Ahmed et al.^28^ Primary technical success was defined as a complete treatment according to protocol with full cover of the lesion by the ablation zone on imaging obtained immediately after ablation.

## Results

### Baseline characteristic

Between April 2017 and October 2022, a total of 85 consecutive patients were included in the study. Patients were 40 (47.1%) men and 45 (52.9%) women with a median age of 68 years (range 31–87 years). There were 96 lesions, located in the bone (36.4%), lung (18.8%), and soft tissue (44.8%) (Table 1). The ablated soft tissue lesions included intra-abdominal masses (37.2%), abdominal wall (18.6%), pelvic masses (16.3%), gluteal masses (7%), adrenal gland metastases (4.6%), and breast nodules (9.3%). Lung lesion size and location, with respect to the pleura, are reported in Table 1. The majority of the treated lung lesions were located <1 cm from the pleura surface (61.2%; 11 of 18). Lesion size was measured by the maximum diameter in the axial image and grouped accordingly. The majority of the lesions were >3 cm in size (44.8%), with 27 lesions (28.1%) measuring 2-3 cm and 26 lesions (27.1%) measuring <2 cm in maximum diameter. The mean lesion size was 3.3 cm, and the median was 2.8 cm. Pelvic bone and rib lesions were the most ablated bone lesions, both 31.5% (11 of 35), and the rest were long bone, shoulder, and others.

**Table 1.** Patient and lesion characteristics.

Patient characteristics	N = 85
Gender, N (%)	
Male	40 (47.1%)
Female	45 (52.9%)
Age (years)	
Median (range)	68 (31-87)
Mean (±SD)	65.87 (12.29)
**Lesion data**	**N = 96**
Location, N (%)	
Bone	35 (36.4%)
Pelvic bone	11 (31.5%)
Rib	11 (31.5%)
Long bone (humerus and femur)	4 (11.4%)
Shoulder Bone	2 (5.7%)
Vertebra	4 (11.4%)
Sacrum	2 (5.7%)
Sternum	1 (2.8%)
Lung	18 (18.8%)
< 1 cm to pleura	11 (61.2%)
> 1 cm to pleura	7 (38.8%)
Soft tissue	43 (44.8%)
Intra-abdominal masses	16 (37.2%)
Abdominal wall	8 (18.6%)
Pelvic masses	7 (16.3%)
Gluteal	3 (7%)
Adrenal gland	2 (4.6%)
Breast nodules	4 (9.3%)
Others	3 (7%)
Lesion size (all)	
< 2 cm	26 (27.1%)
2-3 cm	27 (28.1%)
> 3 cm	43 (44.8%)
N- Number	

### Primary technical success

The overall primary technical success was achieved in 97.6% (83 of 85). Two patients, both with bone lesions, did not undergo cryoablation due to procedural risks as it was impossible to obtain an adequate distance from the proximal structures despite the hydrodissection. For lung and soft tissue lesions, the primary technical success rate was 100%.

### Complications rate and severity

The total reported complications rate within 24 hours of the procedure was 26% (25 of 96) (Table 2), out of which 100% were SIR grade 1 or 2 AEs. Two patients were reported to have asymptomatic pulmonary embolism, noted incidentally with a small (< 1 cm) embolism in a segmental branch of the pulmonary artery in the 24 hours follow-up CT. These patients did not require any oxygen support, while were treated with anticoagulants (SIR grade 2). The most frequent complications were simple (non-hemorrhagic) and hemorrhagic pleural effusions not requiring drainage (SIR grade 1) in 18.7% (18 of 96) (Table 2). Three patients had two AEs related to the same procedure. None required blood transfusion.

**Table 2.** Complications in all cryoablation procedures.

Complication	Number of occurrences (N = 25)	SIR Grade
Hemorrhagic pleural effusion	7	1
Hydropneumothorax^a^	2	1
Hydropneumothorax^b^	2	2
Pleural effusion	11	1
Asymptomatic small pulmonary embolism—incidental findings detected on CT	2	2
Rectus abdominis bleeding^c^	1	2

a Not requiring drainage.

b Requiring drainage.

c Requiring compression bandage.

#### Cryoablation in bone

A total of 35 bone lesions included lesions in the pelvic bone, rib, long bone (ie, humerus and femur), shoulder, vertebra, sacrum, and sternum. Out of those 33 bone lesions ablated, SIR grade 1 pleural effusion was reported in six patients (18.2%; 6 of 33), five of whom had lesions located in the rib and one in the sternum (Table 3). Unfortunately, two patients (6.1%; 2 of 33) with rib lesions ablated were reported as having asymptomatic pulmonary embolisms, with a small (<1 cm) embolism in a segmental branch of the pulmonary artery found incidentally on the 24-hour follow-up CT. These patients did not require oxygen support and were discharged home after a few days (SIR grade 2).

**Table 3.** Complications in bone cryoablation.

Complication	SIR Grade	Number of occurrences (N = 8)	Location (N)
Pleural effusion	1	6 (18.2%)	Rib (5) Sternum (1)
Asymptomatic small pulmonary embolism—incidental findings detected on CT	2	2 (6.1%)	Rib (2)

N—Number; The denominator for all proportions is the number of patients with bone lesions.

#### Cryoablation in lung

**Figure 1.**
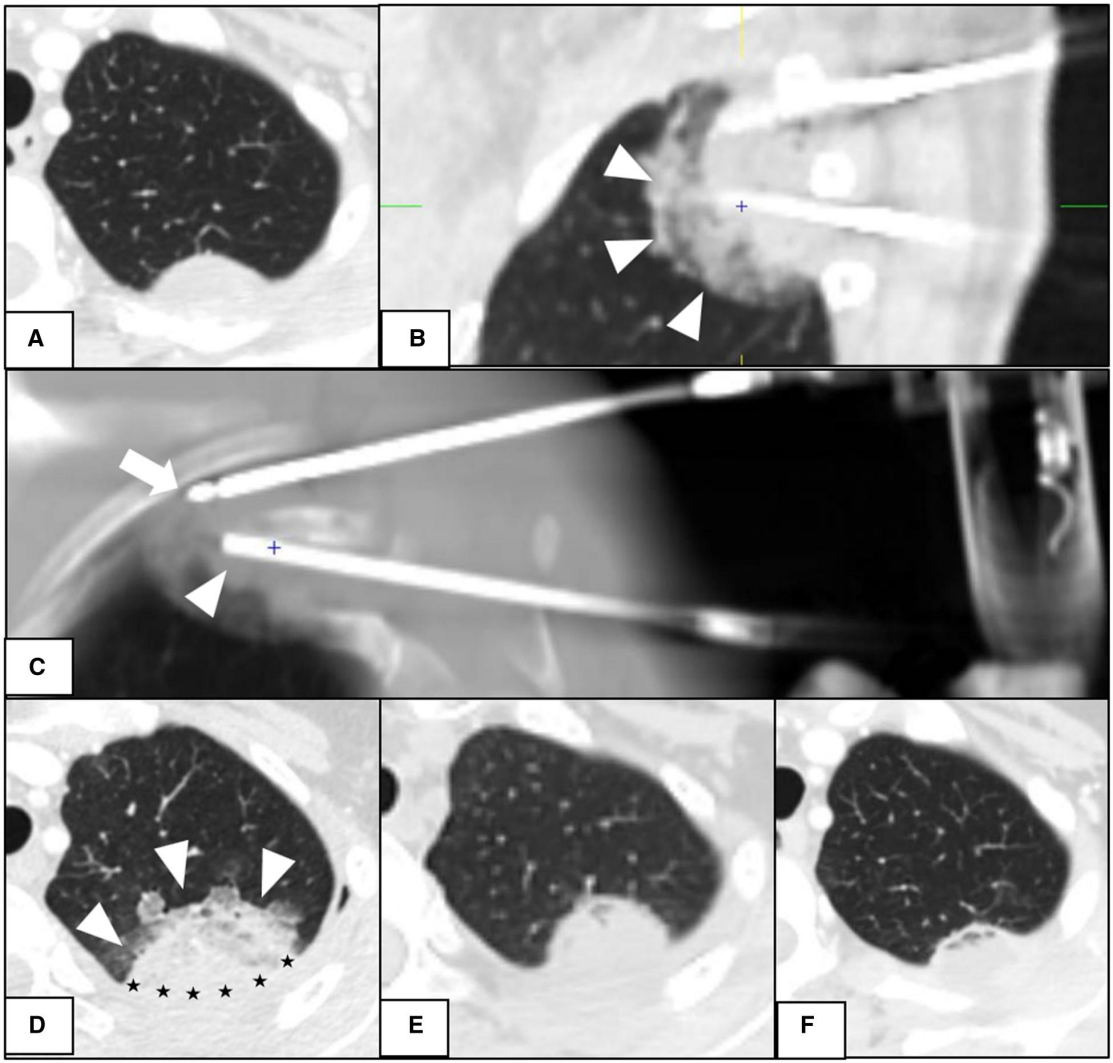
A. lung metastasis, from uterine leiomyosarcoma, with involvement of adjacent pleural layer, relapsed after Radiation Therapy (RT); B. sagittal MPR reconstruction shows the two 10gauge coaxial needle introducers placed into the tumor during cryoablation and the parenchymal effusion surrounding the tumor during treatment (arrowheads); C. Maximum Intensity Projection (MIP) and Multiplanar Reconstruction (MPR) reconstruction shows the cryoprobe (arrow) and cannula (arrowhead) in position; D. parenchymal effusion surrounding the tumor 24 hours later (arrowheads), with reactive pleural effusion (black stars); E. CT scan at 1 month shows initial tumor shrinkage; F. CT scan at 3 months clearly shows further tumor shrinkage. Pleural and parenchymal effusion is no longer visible.

Out of the 18 lung lesions treated, 13 lesions (72.2%; 13 of 18) were reported to have a minor complication (SIR grade 1-2) (Table 4). Nine patients had pleural effusions: three simple (non-hemorrhagic) and six hemorrhagic. Most of these lesions (N = 5) were located at the periphery, < 1 cm in relation to the pleura surface (Figure 1). Symptomatic hydropneumothorax requiring drainage (grade 2) was reported in 2 additional patients. These two patients had central lesions <2 cm in maximum diameter, located > 1 cm from the pleura lining.

**Table 4.** Complications in lung cryoablation.

Complication	SIR Grade	Number of occurrences (N = 13)	Location with respect to pleura (N)	Lesion size (N)
Symptomatic pneumothorax^a^	2	2 (11.1%)	> 1 cm to pleura (2)	< 2 cm (2)
Pneumothorax^b^	1	2 (11.1%)	< 1 cm to pleura (2)	< 2 cm (2)
Hemorrhagic pleural effusion	1	6 (33.3%)	< 1 cm to pleura (3) > 1 cm to pleura (3)	< 2 cm (2) 2-3 cm (2) > 3 cm (2)
Pleural effusion	1	3 (16.7%)	< 1 cm to pleura (2) > 1 cm to pleura (1)	< 2 cm (1) 2-3 cm (2)

N—Number; the denominator for all proportions is the number of patients with lung lesions.

a Requiring drainage.

b Non requiring drainage.

#### Cryoablation in soft tissue

The 43 soft tissue lesions included metastatic intra-abdominal masses, abdominal wall tumor deposits, breast nodules, and adrenal gland tumors. Out of the 4 reported complications, 2 were simple (non-hemorrhagic) pleural effusions (SIR grade 1), in paracardial nodule and adrenal gland ablations, due to the adjacency to the diaphragm. One case of hemorrhagic pleural effusion not requiring drainage (SIR grade 1) was also reported (Table 5). One patient who had treatment for a pelvic mass developed left rectus abdominis muscle bleeding, treated with compression bandage (grade 2).

**Table 5.** Complications in soft tissue cryoablation.

Complication	SIR Grade	Number of occurrences (N = 4)	Location (N)
Hemorrhagic pleural effusion	1	1 (2.3%)	Intra-abdominal masses (1)
Pleural effusion	1	2 (4.7%)	Pericardial nodule (1) Adrenal gland (1)
Left rectus abdominis bleeding	2	1 (2.3%)	Pelvic masses (1)

## Discussion

Cryoablation is an emerging technique in interventional oncology, which induces cellular damage, death, and tissue necrosis through both direct mechanisms involving cold-induced cell injury and indirect mechanisms that alter the cellular microenvironment, thereby compromising tissue viability. This retrospective study is one of the first to use the liquid nitrogen cryoablation technique on patients with tumors in various locations. Overall, percutaneous cryoablation using a liquid nitrogen-based system is shown to be safe for a wide variety of tumor lesions.

We used the latest AE SIR classification system, which received higher ratings in terms of clinical research compared with the existing classification.

In this study, 80% of reported complications were grade 1, with no death-related complications occurring. These results are similar to those reported by a study by Kammoun et al,^4^ who also reported no major complications from cryoablation using a liquid nitrogen-based system in various tumor lesions in bone, kidney, soft tissue, lung, or liver.

In percutaneous cryoablation for metastatic lung cancers, most studies, conducted using an argon gas system, reported pneumothorax as a frequent complication with a rate ranging from 12% to 62%.^8^^,^^22^^,^^29–33^ Comparatively, a recent study by Nomori H et al,^29^ using a liquid nitrogen-base system for the same indication, reported a 25% incidence of pneumothorax (22.6% with Clavien-Dindo grade I). In the current study, a lower rate of pneumothorax requiring drainage in cryoablation of lung lesions (11.1%) was reported. Studies using the argon system (17 gauge cryoprobe) have described higher numbers and larger-diameter cryoprobes as being associated with a higher risk of complications.^31^^,^^34^ In the current study, 10 or 13 gauge cryoprobes were used, depending on the size and location of the lesion, yet the pneumothorax rate remained low (22.2%). Importantly, while the incidence of pneumothorax in this study using a liquid nitrogen-based system was almost the same as in those using an argon gas-based system, the cost of cryoablation with liquid nitrogen is lower. This is due to the typical use of a single cryoprobe compared to the multiple cryoprobes required for argon gas and the lower cost of liquid nitrogen itself compared to argon. Thus, cryoablation with liquid nitrogen is not only as safe as with argon gas but also more cost-effective.^29^

Complications associated with percutaneous cryoablation in soft tissue tumors vary according to their location and proximity to adjacent organs. In our study, lesions included metastatic intra-abdominal masses, abdominal wall tumor deposits, breast nodules, and adrenal gland tumors. For adrenal lesions, studies have reported varied complication rates. For instance, a study by Aoun HD et al,^21^ testing the feasibility and safety of percutaneous cryoablation of adrenal metastases reported a major complication rate (CTCAE > grade 3) of 5%. Another study, a meta-analysis by Pan et al.^35^, reported that 66.9% of complications from adrenal ablation (all modalities) were minor (CTCAE < grade 2) and 33.1% were grade 3 and 4. The most common complication of adrenal ablation was intraoperative hypertension (21.2%), followed by pain (9.49%), post-ablation syndrome, adrenal insufficiency, pneumothorax, and hemorrhages. In our study, only one patient with adrenal lesion developed pleural effusions with no reported major complication or death. This complication was likely due to the proximity of the lesion to the pleural surface and the diaphragm.

A study of safety and efficacy of percutaneous cryoablation of recurrent retroperitoneal soft tissue sarcoma by Fan W et al^36^ reported that patients experienced fever, local pain, emesis, skin frostbite, and nerve injury with rate of 11.4%, 6.6%, 6.0% 3.6%, and 0.6%, respectively, all classified as grade 1 or 2. In comparison, our study found only one patient (2.3%) with a retroperitoneal lesion that developed pleural effusion post-cryoablation.

The overall major complication rate for cryoablation in bone lesions ranges from 0% to 7.4%.^6^ A retrospective study by Auloge P et al^9^ described a total complication rate of 9.1% with no procedure-related mortality. Of these, 2.5% (8 of 320) were major, and 6.6% (21 of 320) were minor complications, suggesting that bone tumor cryoablation is a safe procedure. Minor complications in cryoablation to bone lesions included pain (2.2%), peripheral neuropathy (0.9%), and temporary paraesthesia (0.9%).^9^ Contrary to their study, 18.2% of bone cryoablation cases in our study were reported to have mild simple pleural effusion when the ablated lesions were rib or sternum lesions. These findings were likely related to the location of the lesions, adjacent to the pleural surface. Cryoablation of rib lesions, in particular, will likely cause a reaction to the pleural surface, resulting in reactive simple pleural effusion. Promisingly, pleural effusions were usually self-limiting, and the patients were mostly asymptomatic.

The present study has some limitations, such as the small and heterogeneous study population, including different tumor types and locations, which may introduce conflicting distinct entities, limiting the generalizability. On the other hand, this study may reflect a real-life database, reflecting a diverse patient population, without selection biases. Additionally, some organs were not included in the study (eg, kidney and liver, due to none or limited procedures), and the study focused primarily on complication rates (without long-term efficacy). Future research could encompass a prospective analysis of complications associated with cryoablation in other organs, specifically liver and kidney tumors. Additional studies could assess the local efficacy of tumor treatment at an extended follow-up period.

## Conclusion

In conclusion, cryoablation using the liquid nitrogen-based system is safe across various tumor sizes and locations, with a low rate of minor complications and no deaths reported. The minor complications were self-limiting or required simple interventions like drainage. Factors contributing to complications, such as procedure duration, the learning curve, and medico-economic outcomes, may be the focus of future studies.

## Supplementary Material

tqae171_Supplementary_Data
