# Supplementary material for: Liquid nitrogen-based cryoablation: complication rates for lung, bone, and soft tissue tumors cryoablation
Source: Br J Radiol. 2024 Sep 3;97(1163):1863–9. doi: 10.1093/bjr/tqae171 (PMC11491613; doi:10.1093/bjr/tqae171)
Supplement: tqae171_Supplementary_Data [file tqae171_supplementary_data.docx]

**ARTICLE TITLE**

Liquid nitrogen-based cryoablation: complication rates for lung, bone, and soft tissuetumors cryoablation

**JOURNAL/ TYPE of MANUSCRIPT**

BJR/ FULL PAPER

**MATERIAL AND METHODS**

***Cryoablation procedure and technique***

Cryoprobe was guided by US and CT together using a fusion imaging technique for soft tissues or CT and an electromagnetic navigation device for lung and bone treatment.

Cryoablation generally involves a double 10-minute freeze protocol consisting of two 10-minute freeze cycles separated by a 10-minute passive thaw [8, 10, 22]. Intermittent CT images were taken to monitor the size of the iceball, ensuring it expanded at least 5-10 mm beyond the tumor borders. The duration of the cycles could be shortened at the operator's discretion if the iceball approached a critical structure or if the thermos-protective measures, such as hydro-dissection, were insufficient for a safe procedure. Additional freezing cycles and repositioning or replacement of a cryoprobe were performed if the iceball did not achieve the required margins.

Thermo-protective measures, including hydro-dissection and/or thermocouple placement, were employed, when necessary, to protect non-targeted areas such as the skin in superficial lesions or nerve roots. Hydro-dissection involved injecting saline through the 21 gauge needle between the iceball and the adjacent vulnerable organ to displace or insulate the organ.
